# Fine-scale genetic differentiation in the bee-specialized *Antirrhinum charidemi* covaries more strongly with spatial isolation than with corolla colour

**DOI:** 10.1093/aobpla/plaf017

**Published:** 2025-03-24

**Authors:** Myriam Heuertz, Marcial Escudero, José María Gómez, Pablo Vargas

**Affiliations:** Univ. Bordeaux, INRAE, BIOGECO, 69 route d’Arcachon, F-33610 Cestas, France; Department of Plant Biology and Ecology, University of Seville, Reina Mercedes 6, E-41012 Seville, Spain; Estación Experimental de Zonas Aridas (EEZA-CSIC), Ctra Sacramento s/n, La Cañada de San Urbano, E-04120 Almería, Spain; Research Unit Modeling Nature (MNat), Universidad de Granada, E-18071 Granada, Spain; Real Jardín Botánico, RJB-CSIC, Plaza de Murillo 2, E-28014 Madrid, Spain

**Keywords:** *Antirrhinum charidemi*, bee specialization, flower colours, genetic structure, nuclear SSRs, pink morph, plastid haplotypes, spatial isolation, white morph, topography

## Abstract

The snapdragon *Antirrhinum majus* has been a model species for genetics, plant development, and evolution since the 19th century. Recent studies have expanded the focus to the entire *Antirrhinum* genus as a model system for rapid evolution (26 species in < 5 million years). However, in-depth studies to reconstruct microevolution in additional snapdragon species are lacking. This study aimed to explore to what extent potential pollinators, flower colour morphs, spatial and environmental factors contribute to differentiation in a small population of the Mediterranean *A. charidemi* (south-eastern Spain). We studied a population of approximately 200 *A. charidemi* individuals with either pink or white corollas, characterized by strong topographic heterogeneity (horizontal extension of 120 × 80 meters; 40-meter altitude difference) and diversity in environmental factors (substrate, vegetation). The study analysed pollinator preference for either white or pink corollas, genetic diversity using 13 nuclear SSR loci and three plastid haplotypes, and the spatial population structure. Flower visitors displayed some indication of preference for pink corollas (five of ten bee species) and flower colour morphs were genetically differentiated. However, the strongest pattern of genetic differentiation was associated with a fine-scale spatio-topographic isolation in the population, with five topo-genetic subpopulations and a pollen-to-seed dispersal distance ratio of 4.32. Our results agree with similar patterns of strong spatial genetic isolation found in *A. charidemi* at larger scales: phylogeographic differentiation of populations and phylogenetic relationships within a south-eastern Iberian *Antirrhinum* clade. Despite the extreme corolla specialization for bee pollination, spatial isolation appears to be the predominant factor driving short- and long-term differentiation in *A. charidemi*. We argue that a comprehensive understanding of early stages of rapid evolution requires detailed investigation of fine-scale evolutionary drivers, including both spatial isolation (topography) and ecological factors (e.g. pollination fauna).

## Teaser

This study explores the factors contributing to genetic differentiation in a small population of the Mediterranean snapdragon Antirrhinum charidemi, an endemic of the Cabo de Gata region in Southern Spain. The population, characterized by topographic heterogeneity and environmental diversity, includes individuals with either pink or white corollas. While insect flower visitors showed a slight preference for pink corollas, and flower colour morphs were genetically differentiated, the most significant genetic differentiation was driven by fine-scale spatial and topographic isolation. The study discusses the importance of spatial isolation in both short- and long-term differentiation in the genus Antirrhinum, despite its specialised bee-pollination. The findings suggest that a detailed investigation of fine-scale evolutionary drivers, including topography and ecological factors, is crucial for understanding the early stages of rapid evolution in *A. charidemi* and similar species.

## Introduction

In most angiosperms, flower colouration has historically been attributed to a decisive role in driving speciation through pollinator-mediated reproductive isolation (Narbona *et al.* 2021). Indeed, we can find a wide range of colour patterns among flowering plants. They range from large clades of species with a single colour morph (e.g. white, purple, or yellow in *Cistus*, Guzmán and Vargas 2005) to two or more colour morphs within the same population of a single species (Streisfeld *et al.* 2013; Del Valle *et al.* 2019; Jiménez-López *et al.* 2023). However, it has been argued that pollinators are not always responsible for evolutionary transitions in flower colour variation (Rausher 2008). A recent review showed that there is little evidence for significant pollinator-mediated selection among existing studies (Trunschke *et al.* 2021). Colour transitions unrelated to pollinator-mediated selection can result from climatic variation, antagonist-mediated selection, genetic drift, pleiotropic effects, or genes influencing anthocyanin pathways in vegetative rather than reproductive traits (Rausher 2008; Koski and Ashman 2016; Smith 2016; Del Valle *et al.* 2019).

Anthocyanins play a crucial role in plant physiology, providing antioxidant functions and protecting plants from various biotic and abiotic stresses, but are also key pigments responsible for the diverse hues of red, purple, and blue in plant organs like fruits and flowers (Torres *et al.* 2002; Landi *et al.* 2015; Mannino *et al.* 2021). The occurrence of anthocyanins associated with purple flowers is commonplace in many floras. For instance, the flora of the Mediterranean floristic region (around 25,000 species) has a high percentage (87.7%) of species with flower colour polymorphisms (Kantsa *et al.* 2017; Narbona *et al.* 2018). In the Mediterranean basin, snapdragons (species of *Antirrhinum*, Plantaginaceae) form a plant group that exhibits one of the highest levels of colour polymorphism of flowers. *Antirrhinum majus* L. has been historically used as a model plant in genetics and flower development (Schwarz-Sommer *et al.* 2003), including studies of flower colour (purple, yellow, white) transitions at the populational level (Whibley *et al.* 2006; Bradley *et al.* 2017; Tavares *et al.* 2018). Despite the well-studied role of colouration in *Antirrhinum majus*, the evolutionary significance of flower colour variation in other *Antirrhinum* species remains unclear.

**Figure 1.**
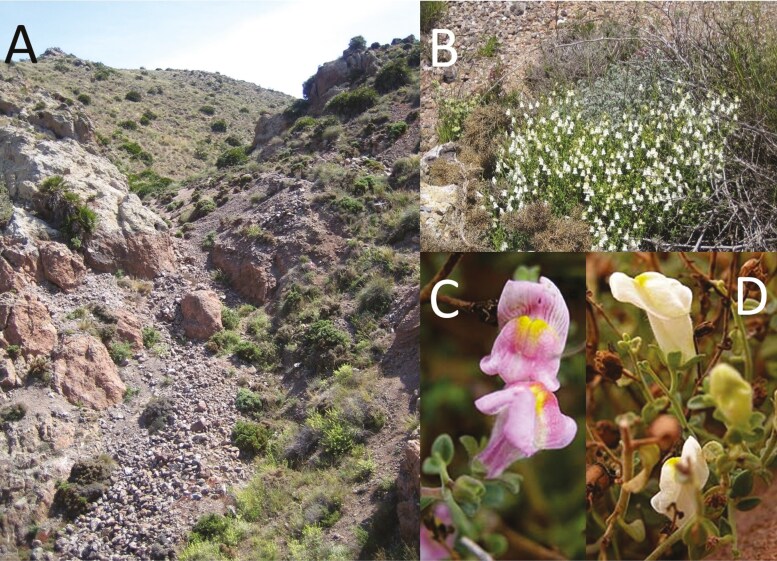
Studied population of *Antirrhinum charidemi* in the *Barranco del Dragoncillo Blanco* (Almería, Spain). View of the ravine where the c. 200 individuals occurred (2010) (A); one of the 10 individuals with white corollas (B); close-up of the two corolla types found on each individual showing the morphs pink (C) or white (D). Photo credit: Myriam Heuertz (A, B) and Pablo Vargas (C, D).

In addition to colouration, flower shape has been proposed as a strong physical barrier to pollinators of all the species of *Antirrhinum* (Sutton 1988; Magallón and Vargas 2014). Indeed, the constant flower phenotype of snapdragons displays one of the most occluded corollas among angiosperms (Fig. 1), which is only accessible by bees (Torres *et al.* 2002; Vargas *et al.* 2010, 2017; Magallón and Vargas 2014). As a result, a tight mutualism between snapdragons and bumblebees has been historically proposed (Macior 1967; Odell *et al.* 1999; Dyer *et al.* 2007; Whitney *et al.* 2013). Once bee pollination was confirmed, researchers investigated in detail the relationship among occlusion, corolla colour variation, and pollination by bumblebees in *Antirrhinum majus* variants in order to elucidate the genetic pathways underlying the evolution of these floral traits (Whibley *et al.* 2006; Shang *et al.* 2011; Bradley *et al.* 2017; Tavares *et al.* 2018; Qiao *et al.* 2023). Numerous feeding experiments using *A. majus* have demonstrated that: (a) *Bombus* species discriminate flower colouration (Comba *et al.* 2000); (b) *Bombus terrestris* associates warmth with corolla floral colour (Dyer *et al.* 2007); (c) single mutations affecting flower signal (colour, reflectance) can have profound effects on pollinator behaviour (Dyer *et al.* 2007; Moss *et al.* 2024); (d) a corolla with conical epidermal cells can provide fitness benefits (Glover and Martin 1998; Whitney *et al.* 2011); e) the corolla venation patterning might have evolved as a response to bees learning of their use as nectar guides (Whitney *et al.* 2013; Tavares *et al.* 2018). To link the evolutionary patterns between *A. majus* and relatives sharing the distinctive occluded flower shape, numerous studies have contributed to a broader understanding of this unique floral morphology, which appears to have emerged between 30 and 45 million years ago (Vargas *et al.* 2009, 2014; Gorospe *et al.* 2020).

Taxonomic treatments of *Antirrhinum* considered morphological characters including flower colours to group the approximately 26 currently accepted species into three subsections: Antirrhinum (11 species with purple, magenta, and yellow corollas); Streptosepalum (two species with yellow corollas); and Kickxiella (13 species displaying mainly whitish flowers) (Rothmaler 1956; Otero *et al.* 2021). Phylogenetic results do not support these groupings and a more dynamic transition of corolla colours appears to have occurred in the course of evolution of *Antirrhinum* (Otero *et al.* 2021; Durán-Castillo *et al.* 2022). At a macroevolutionary scale, the white corolla is the predominant phenotype in the majority of *Antirrhinum* species, which are intermingled in the three main clades (Otero *et al.* 2021; Durán-Castillo *et al.* 2022). In contrast, a predominant pattern of geographic speciation in *Antirrhinum* was inferred using both genetic (Vargas *et al.* 2009) and genomic (Otero *et al.* 2021) data. The question remains as to what extent white corollas have been favoured at early stages of population differentiation (microevolutionary scale).

Based on more than 30 studies on *Antirrhinum*, two non-exclusive hypotheses are proposed to account for the rapid differentiation (26 species) in the last five million years (Vargas *et al.* 2009). On the one hand, a tight snapdragon–bumblebee dependence in self-incompatible species (Vargas *et al.* 2017) indicates that bees may have triggered shifts in flower morphology and corolla colouration in the course of evolution. On the other hand, the narrow distribution of a considerable number of snapdragons (9 species) across the Iberian Peninsula (Güemes 2009; Vargas *et al.* 2009; Vargas 2024), together with a biogeographic pattern for the whole genus (Otero *et al.* 2021), suggests that geography has been key for speciation. The two hypotheses were addressed in an analysis of pollinators and geographic distribution of the species, primarily 11 bumblebee species across 18 species of *Antirrhinum* across the Iberian Peninsula (Vargas *et al.* 2017), suggesting that both hypotheses were likely at play. The extent to which early stages of evolutionary divergence (i.e. population differentiation) are primarily driven by pollinator preference for flower colouration or by spatial isolation is, however, unclear. In other words, is isolation by ecology (pollinator preference) or isolation by distance (spatial differentiation) predominantly responsible for triggering early genetic differentiation within *Antirrhinum*?

To address the question of the drivers of incipient evolutionary differentiation, we examined flower colouration, pollinator behaviour, and population genetic structure in a topographically complex population formed by plants with either pink or white corollas of a narrow-endemic snapdragon species, *Antirrhinum charidemi* Lange in southeastern Spain. In particular, we addressed the following hypotheses:

- Hypothesis 1: Under a scenario of pollinator preference for flower colour, pollinators are expected to discriminate by corolla colouration and will thus preferentially visit and pollinate plants of their favoured colour morph. Over time, assortative mating due to pollinator preference is expected to lead to genetic divergence patterns associated with individuals with a particular flower colour (Rymer *et al.* 2010).
- Hypothesis 2: Alternatively, under a spatial isolation scenario, early genetic differentiation is associated with distance and topography rather than flower colouration.
- Hypothesis 3: Since genetic structure is not only shaped by patterns of pollen dispersal (mediated by pollinators) but also by spatially limited seed dispersal (barochory, wind), complementary information on the relative roles of both dispersal vectors can be gained by examining genetic structure and gene dispersal estimates from maternally and biparentally inherited markers (plastid sequences vs. SSRs).

In addition, some other ecological characteristics were addressed to test the contribution of local conditions such as altitude, substrate, and co-occurring vegetation.

## Materials and methods

### Study species and focal population

**Figure 2.**
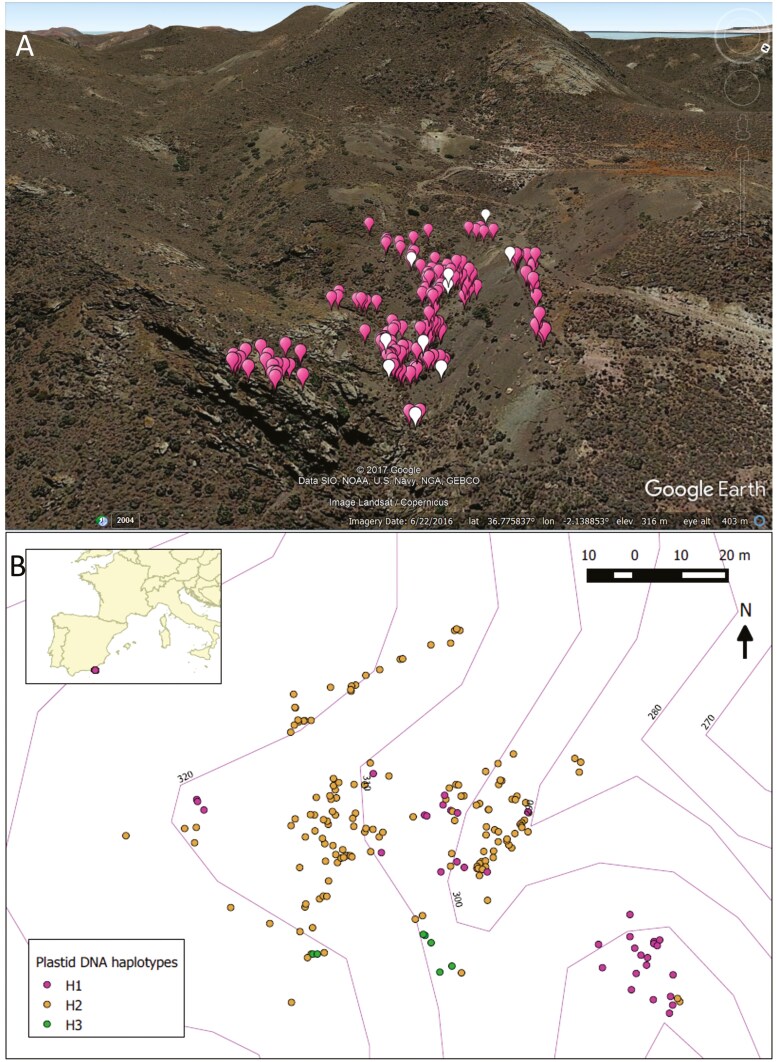
Spatial distribution of adult plants of *Antirrhinum charidemi* in the BDB (*Barranco del Dragoncillo Blanco*) population in 2009-2010. A. Satellite image from Google Earth: white-flowered individuals are represented by white symbols and pink-flowered individuals by pink symbols. ©2017 Google Data SIO, NOAA, U.S. Navy NGA, GEBCO, Image Landsat/Copernicus, Imagery date 6/22/2016, lat. 36.78º, lon -2.14º, elev. 316m, eye alt. 403 m. B. Distribution of plastid DNA haplotypes (H1, H2, H3) represented on a terrain model in QGis. Note that the direction of North is not the same in A and B.

*Antirrhinum charidemi* Lange is a perennial shrub endemic to an 8 × 4 km region in the volcanic mountains of Cabo de Gata (Almería, south-eastern Spain), where populations are formed by few to hundreds of individuals (Forrest *et al.* 2017). The flower colour is generally pink, with weak variation in colour intensity. Some flowers are observed all year round in certain individuals, although all individuals are flowering between March and June (Cueto *et al.* 2008; Vargas *et al.* 2010; M. Heuertz, personal observation). We focussed our study on the Barranco del Dragoncillo Blanco (BDB, 36°46′55′′ N, 2°07′54′′ W) population of ca. 200 individuals distributed in a ravine (*barranco* in Spanish), on rocky outcrops and on scree slopes bordering the ravine (Figs. 1A and 2). This is the population where the highest genetic diversity was found in *A. charidemi* (Forrest *et al.* 2017). We visited the area regularly between 2009 and 2010. The flowering peak in 2009 was reached in May, including 10 individuals displaying white corollas (Fig. 1B) and 172 individuals displaying pink corollas (Fig. 1C).

### Flower colour variation

The five petals of any *Antirrhinum* species display a personate flower, i.e. the corolla has a tube, an adaxial or upper lip (two fused upper petals), and a lower or abaxial lip (three fused lower petals) that typically develops a basal convexity (palate) strongly occluding the mouth of the tube (see Vargas 2024 at www.rjb.csic.es/snapdragons) (Fig. 1C and D). All the flowers of *A. charidemi* display the same occluded corolla shape and no size differences between pink and white corollas were observed (typically corolla tubes are about 19 mm long and 6 mm wide; see Vargas *et al.* 2010). Both colour morphs have yellow spots on the tube sides and the lower lip where it touches the upper lip (corolla access) (Fig. 1C and D). Every flower lasts 5–7 days (P. Vargas personal observation) and produces one of the highest amounts of sugar (4.5 mg per flower in 24 h) among the flowers of eastern Andalusia (Herrera 1988). Reflection of UV light was measured for the flowers of two individuals of each colour morph, using an USB4000 miniature fiber optic spectrometer with a USB-DT deuterium tungsten halogen source (Ocean Optics, Dunedin, Florida, USA).

### Preference pattern of pollinators for flower colour

To test whether pollinators exhibited any preference pattern for flower colour (Hypothesis 1), we conducted a pollinator observation experiment. During 2007, we established three subplots, in each of which we selected one *A. charidemi* individual with white and one of similar size with pink corollas growing side-by-side. During the main flowering period (March to June) we examined for 15-minute periods (trials hereafter) all insects visiting the flowers of the experimental plants, counting only those that successfully opened the corolla and contacted the reproductive organs. Although no direct tests were conducted to estimate the success of pollen deposition in visited flowers, we considered that flower visitors (typically bees) are likely actual pollinators of *A. charidemi*. This consideration was based on (i) low flower visit ratio by insects other than bees in *Antirrhinum* (Vargas *et al.* 2017), (ii) high efficiency of bees in specialized flowers (Fenster *et al.* 2004), and (iii) high levels of fruit and seed sets when bees touched flower reproductive parts with their large body sizes and suitable structures (hairy scutum) (Vargas *et al.* 2010). Visiting insects were identified using local faunas and taxonomic treatments (Ornosa and Ortiz-Sánchez 2004; Michener 2007; Vargas *et al.* 2010). To control for the difference in flower availability, the number of flowers on the experimental plants was counted during each trial. We performed all trials on sunny days, with a temperature of over 20°C and without wind. We also visited populations of the species when weather conditions were different (rain, wind, low temperature) and hardly observed insects visiting the plants (see Vargas *et al.* 2017). In total, we conducted 140 trials, with a total of 2,100 minutes (35 h) of observation.

The preference of each insect visiting the flowers of the experimental plants was estimated using the Jacobs’ D index (Jacobs 1974; Lechowicz 1982). This index compares the relative availability of each flower colour and their relative visits by each pollinator, taking a value of zero under random feeding and deviating symmetrically from zero between 1 and -1 for preferred and avoided items, respectively. Jacob’s D index was calculated using the function *ivlev* in the R package *selectapref* (Richardson 2020). The statistical significance of the preferences was obtained by testing for deviation from random visitation rate using a test for goodness of fit (Lechowicz 1982). Under random visitation, the null hypothesis is that the proportion of insects visiting a given flower colour equals the proportion of the flower colour in the environment (Lechowicz 1982). In addition, to analyse if flowers with different colour attract different potential pollinators, we tested whether the composition of the visiting insect assemblage varied between plants with different flower colours by means of a Permutational Analysis of Variance (Anderson 2001). This analysis was performed using the function *adonis2* in the R package *vegan* on a Bray-Curtis dissimilarity matrix (Oksanen *et al.* 2001).

### Sampling of plant material and ecological data collection

Leaf samples were collected from 182 *A. charidemi* individuals at BDB between 2009 and 2010 and samples were silica-dried. The sampling process involved meticulously scouting the studied area where plants were located within ca. 120 × 80 m, with 40 m difference in altitude, and its bordering area. For each individual, we recorded geographic position and altitude using a Topcon geospatial station (mapping ca. 80% of the individuals with a precision of 2 mm for distances up to 500m) or a handheld GPS device (Garmin Oregon, used in subsequent field visits with multiple position measurements per individual to reach a GPS precision of ca. 2m). We also recorded plant height and diameter, as well as vegetation at immediate contact with the target plant possibly preventing herbivory and providing a favourable microenvironment for germination. DNA was extracted from ca. 15 mg of dried leaf tissue using the Invisorb DNA Plant HTS 96 Kit (Invitek, Berlin, Germany).

### Nuclear microsatellite analyses

We amplified 14 microsatellite (SSR) loci in two multiplexes using the QIAGEN multiplex PCR kit (Qiagen) following the manufacturer’s protocol, as described in Forrest *et al.* (2017). Forward primers were fluorescently labelled and fragments were resolved on an ABI3730 DNA analyzer (Applied Biosystems). Allele sizes were determined using Genemapper 3.7 (Applied Biosystems) by comparison with the GeneScan™ -500 LIZ size standard.

### Plastid DNA analyses

Two plastid DNA (pDNA) spacers (*trn*D-T and *trn*S-fM) were chosen based on Forrest *et al.* (2017), PCR amplified and sequenced in all individuals using the services of Macrogen (dna.macrogen.com). Sequences were assembled and edited with CodonCode Aligner ver. 3.7.1 (CodonCode Corporation, MA).

### Genetic data analyses

#### Genetic diversity

To assess SSR marker quality we checked for null alleles using Micro-checker version 2.2.3 (van Oosterhout *et al.* 2004) and assessed linkage disequilibria between loci using Genepop version 4.7.5 (Rousset 2007), correcting for multiple testing with a sequential Bonferroni correction. We estimated genetic (pDNA and SSRs) diversity parameters for the 182 individuals of the BDB population using the software SPAGeDi ver. 1.5a (Hardy and Vekemans 2002), as well as for groupings according to (i) flower colour or (ii) geo-genetic subpopulations, defined as geographically delimited (spatio-topographic) subpopulations in which individuals generally shared genetic cluster membership (see STRUCTURE analysis described below). For pDNA, we assessed allelic (haplotype) richness using rarefaction, AR, and gene diversity, *H*_E_, corrected for sample size. For SSRs, we estimated AR, *H*_E_, and the individual inbreeding coefficient *F*_IS_ and tested for deviation from Hardy-Weinberg genotypic proportions using permutation tests (10 000 permutations).

#### Determinants of within-population genetic structure

To assess the effect of ecological predictors on genetic differentiation within BDB, we used a matrix-based modelling approach that allowed us to evaluate the effect of flower colour (through pollinator preference, Hypothesis 1), the effect of spatial isolation (Hypothesis 2), and effects of additional predictors, alone or in combination. Genetic similarity between pairs of individuals was assessed by means of the kinship coefficient *F*_ij_ (Loiselle *et al.* 1995) between individuals i and j using SPAGeDi. The nuclear kinship matrix was set as the response variable (the plastid matrix was not used because of too low variation). We ran eleven models using five predictor variables and combinations thereof using the Bayesian generalized linear mixed modelling approach implemented in the *MCMCglmm* package (Hadfield 2010) in R: (i) NULL, a null model without any predictor, (ii) GEO, with a predictor matrix representing spatial distance between individuals, computed from easting and northing (latitude and longitude), (iii) ALT, with a distance matrix based on altitude only, (iv) SUB, with a matrix based on sharing topography (ravine vs. scree slopes/outcrops, pairs of individuals on the same substrate are coded as ‘1’; pairs on distinct substrates as ‘0’), (v) COL, with a matrix based on sharing of corolla colour, ()vi) VEG, with a matrix predictor based on sharing vegetation at individual plant contact, i.e. conditions potentially facilitating seedling establishment in harsh environmental conditions. To construct this matrix, we assessed whether individuals were in immediate contact with other plants; 16% missing data were imputed using the *mice* package (van Buuren and Groothuis-Oudshoorn 2011) in R. We also applied the following models with combinations of these matrices. (vii). GEO_ALT, 8. GEO_ALT_SUB, 9. COL_VEG, 10. GEO_ALT_COL, 11. GEO_COL matrices. MCMCglmm was initiated with standard priors and run with a burn-in of 5000 followed by 20 000 iterations with a thinning interval of 750. Model comparison was conducted by means of comparing the Deviation Information Criterion, DIC.

#### Characterization of within-population genetic structure

To test for spatial autocorrelation of genetic relatedness in each plastid and nuclear data, the matrices of inter-individual kinship coefficients (see above) were regressed on the logarithm of geographic distance to yield regression slopes, *b*_p_ for plastid and *b*_n_ for nuclear DNA. These slopes were compared to their expected distributions obtained from 10 000 permutations of rows and columns of the spatial distance matrix. To quantify the strength of SGS, we computed *Sp* statistics (*Sp*_p_ for plastid and *Sp*_n_ for nuclear DNA) from the regression slopes as –*b*/(1 − *F*_1_), where *F*_1_ is the average kinship coefficient for ‘neighbouring’ individuals, i.e. for all pairs of individuals in the first distance class (Vekemans and Hardy 2004). Seven distance classes were defined for computation of *F*_1_ and graphical representation, with maximal inter-individual distances of 3, 6, 12, 24, 48, 96, and 192 m.

To additionally explore non-linear SGS patterns, we used a spatial principal component analysis (sPCA) in the *adegenet* package (Jombart 2008) in R. Spatial genetic autocorrelation (e.g. patches of related individuals, allele frequency gradients) was tested using a G-test, whereas inter-individual genetic repulsion was tested with an L-test. The strength of SGS was estimated as the eigenvalue of the first sPCA axis, eig.sPCA.

We further used genetic clustering methods; specifically, the STRUCTURE (Pritchard *et al.* 2000) and GENELAND (Guillot *et al.* 2005) programmes, respectively ignoring or including the spatial position of individuals as prior information. STRUCTURE ver. 2.3.2.1 (Pritchard *et al.* 2000) was run using the admixture model with correlated allele frequencies and *K* = 1 to *K* = 12 genetic clusters. For each *K*, ten runs were performed with 100,000 iterations discarded as burnin, followed by 500,000 iterations. Analyses were parallelized on the Genotoul computer cluster, INRAE Toulouse, France, using the StrAuto software (Chhatre and Emerson 2017). The *K* that best explained the data was inferred using maximization of lnP(data) and the deltaK criterion in StructureHarvester (Earl 2012). STRUCTURE results were combined across runs using Clumpak (Kopelman *et al.* 2015) and the ancestry proportions of each individual in each gene pool were mapped as pie charts and using spatial interpolation (Inverse Distance Weighting) with QGis Desktop (QGIS 2017). In GENELAND (Guillot *et al.* 2005), five MCMC chains were run using a spatial model with correlated allele frequencies, simulating K populations in Hardy-Weinberg equilibrium and unlinked loci (allowing K from 1 to 20; Guillot *et al.* 2005; Guillot 2008). We used 100,000 MCMC iterations with a thinning of 100 and setting the burn-in to 20,000 iterations.

To comparatively address the support of our data to Hypotheses 1 and 2, we computed genetic differentiation as *F*_ST_ between flower colour morphs, among STRUCTURE gene pools assigning individuals to gene pools based on *q* > 0.5 gene pool membership, and among geo-genetic subpopulations to which individuals were assigned based on their spatio-topographic position and the results from STRUCTURE.

#### Inference of gene flow patterns

To address Hypothesis 3 on the intra-population gene flow and its components due to seed and pollen dispersal, we first used coalescent simulations and Bayesian inference in the program MIGRATE (Beerli 2008), and second, the *Sp* estimates from SPAGeDi. For the MIGRATE analysis, we considered the geo-genetic subpopulations previously defined. We assumed a constant mutation rate across loci for the mutation-scaled effective population size Θ and ran migration matrix models with a variable Θ and, average (model ‘mean_m’), symmetric (model ‘symmetric_m’) or freely variable (model ‘free’) migration rates between all combinations of subpopulations. We also implemented models with a fix population parameter Θ and an average (model ‘fixtheta_mean’) or symmetric (model ‘fixtheta_sym’) migration rates. We used uniform-wide priors and ran two replicates of one long Markov chain with 10 000 steps discarded as burnin and 5000 steps retained for estimates, with a sampling increment of 100 steps. The heating scheme was static with four chains of distinct temperatures (1, 1.5, 3, 1,000,000), swapping at each step. Model choice was conducted using Bayes Factors based on the Bezier approximated score for Log-Probability of the data given the model. Estimates of migration rates were plotted on the population map using the Flowmapper plugin (Gulluoglu 2016) in QGIS Desktop.

We further estimated historical dispersal distances from *Sp* under the assumption of drift-dispersal equilibrium in the population. In hermaphroditic plants, the *Sp* statistics are expected to approach 1/(4π*D*_*e*_*σ*_*g*_^2^) for nuclear (biparentally inherited) and 1/(2π*D*_*e*_*σ*_*s*_^2^) for plastid (maternally inherited) markers at equilibrium, where *σ*_*g*_^2^ is half the mean squared dispersal distance of genes, *σ*_*s*_^2^ is half the mean squared dispersal distance of seeds, and *D*_*e*_ is the effective population density (Vekemans and Hardy 2004). Thus, *Sp* computed separately from nuclear and plastid markers can provide information on the relative distances of gene vs. seed dispersal, and by inference, of the pollen to seed dispersal ratio. We used *D*_*e*_ = 0.01 individual per m^2^, corresponding to half the census population density, thus assuming a relatively balanced contribution of individuals to reproduction. This value was justified by the long flowering season and observation of flowers in all adults. We used an iterative procedure for the estimation of *σ*_*g*_ and *σ*_*s*_ from the kinship-ln(distance) regression, in order to estimate these statistics from the appropriate distance range (Heuertz *et al.* 2003). The relative contribution of seed dispersal to overall gene dispersal is then given by the *σ*_*g*_*/σ*_*s*_ ratio and applying *σ*_*g*_^2^ = *σ*_*s*_^2^ + ½ *σ*_*p*_^2^ (Crawford 1984), we obtained the pollen-to-seed dispersal distance ratio as *σ*_*g*_/*σ*_*s*_.

## Results

### Corolla features and pollinator behaviour

The spectral profiles of the flowers were remarkably similar between pink and white corollas, with no ultraviolet signals detected in any of the samples [Supplementary Information—Fig. S1]. We recorded a total of 878 visits by ten bee species (*Apis mellifera*, *Anthophora plumipes*, *Anthophora dispar*, *Eucera alternans*, *Ceratina cucurbitina*, *Megachile deceptoria*, *Rhodanthidium sticticum*, *Chalicodoma lefebreyi,*  *Chalidocoma sicula*, and *Osmia submicans*) and one beetle species (*Oxytyrhea funesta*) entering the flowers of the observed plants (Table 1). Altogether, the visiting insects assemblage significantly preferred to visit pink flowers than white flowers (Table 1), indicating support for our Hypothesis 1. This preference for pink flowers remained the same when splitting the visiting insects into two groups: territorial and non-territorial species (Table 1). In addition, three of the five species with sufficient sample size also showed a significant preference for pink flowers, whereas the other two (*Rhodantidium sticticum* and *Osmia submicans*) did not prefer to visit any floral morph (Table 1). Flower colour preference by bee species, including results of the Jacob´s D index and proportion of corolla colour preference, are shown in Supplementary Information—Figs. S2 and S3, respectively. The permutation-based multivariate analysis of variance (PERMANOVA) for the two corolla morphs showed similar relative frequencies of visits by bee species [Supplementary Information—Table S1].

**Table 1.** Preference patterns *of A. charidemi* pollinators.

	Number of insect visits		Visitation rate (Visits per flower per day)		Jacob’s D index of preference			
Pollinators (T, territorial or floral constancy; N, non-territorial)	White morph	Pink morph	White morph	Pink morph	White morph	Pink morph	Chi- square	*P*-value
HYMENOPTERA								
*Apis mellifera* (T)	3	0	0.003	0.000	1.00	−1.00	4.95	0.026
*Anthophora plumipes* (T)	3	6	0.003	0.003	−0.10	0.10	0.08	0.784
***Anthophora dispar* (N)**	**9**	**42**	0.008	0.022	−0.48	0.48	**8.78**	**0.003**
*Eucera alternans* (N)	0	3	0.000	0.002	−1.00	1.00	1.82	0.177
***Ceratina cucurbitina* (T)**	**9**	**59**	0.008	0.032	−0.60	0.60	**17.40**	**0.000**
*Megachile deceptoria* (N)	3	0	0.003	0.000	1.00	−1.00	4.95	0.026
***Rhodanthidium sticticum* (T)**	**72**	**144**	0.063	0.077	−0.10	0.10	**1.80**	**0.180**
***Chalicodoma lefebreyi* (N)**	**133**	**308**	0.117	0.165	−0.17	0.17	**10.83**	**0.001**
*Chalicodoma sicula* (N)	3	0	0.003	0.000	1.00	−1.00	4.95	0.026
***Osmia submicans* (N)**	**26**	**54**	0.023	0.029	−0.12	0.12	**0.94**	**0.332**
COLEOPTERA								
*Oxythyrea funesta* (T)	1	0	0.001	0.000	1.00	−1.00	1.65	0.199
**Territorial species**	**87**	**209**	0.077	0.112	−0.19	0.19	**8.81**	**0.003**
**Non-territorial species**	**171**	**407**	0.151	0.217	−0.18	0.18	**16.42**	**0.000**
**All species**	**262**	**616**	0.231	0.329	−0.18	0.18	**23.41**	**0.000**

Total number of insects visiting the flowers of the observed plants, visitation rate calculated as number of insects visiting each flower per day, magnitude of Jacob´s D index of preference and results of the goodness of fit testing the statistical significance of the preference pattern are shown. In **bold** those species and sample groups in which tests had a robust sample size. Pollinator´s names are followed by pollination behaviour in brackets (T, territorial or floral constancy; N, nomadic) (see Vargas *et al.* 2017).

### Genetic diversity

Plastid DNA spacer sequences displayed one point mutation each, A or C at the matrix position 137 in *trn*D-*trn*T and T or G at position 35 in *trn*S-*trn*fM, which resulted in three haplotypes (H1: AT, H2: AG, H3: CG). Haplotype data were obtained for 176 individuals (Fig. 2). Haplotype diversity statistics are reported in Table 2. All white-flowered individuals shared the same haplotype, while plants with pink flowers had the three haplotypes. SSR genotypes were obtained for 182 individuals and 13 loci; locus MSAT56 could not be interpreted as a biparentally inherited locus and was therefore excluded. Null alleles were observed at low frequencies at three loci [Supplementary Information—Table S2] and one pair of loci (MSAT55-MAAC4) was in linkage disequilibrium (LD). In the full dataset of 13 loci, 62 alleles were observed, overall genetic diversity was H_E_ = 0.462, with little variation between categories based on flower colour or geo-genetic (STRUCTURE-based) subpopulations (Table 3). The inbreeding coefficient *F*_I_ was not significantly different from zero within categories or subpopulations, as expected for a self-incompatible plant species (Navascués *et al.* 2010). The full dataset of 13 loci was kept for all analyses because removing four loci to correct for null alleles and LD resulted in very similar genetic estimates [Supplementary Information—Tables S3, S4].

**Table 2.** Plastid DNA diversity. *n*, sample size, *NA*, number of alleles, *A*_R_ (*k* = 7) allelic richness in sample of seven gene copies (individuals), *H*_E_, gene diversity corrected for sample size).

Category	*n*	% missing	*NA*	*A*_R_(*k* = 7)	*H*_E_
White	10	0	1	1	0.000
Pink	172	3.5	3	2.02	0.364
All	182	3.3	3	1.99	0.348

**Table 3.** Genetic diversity statistics of SSR markers for flower-colour-based categories, and topographic-genetic groups based on the STRUCTURE analysis.

Category	*n*	missing %	NA	*A*_R_(k = 14)	*H*_E_	*F*_I_	*P*
White	10	0.8	2.92	2.65	0.387	0.056	0.496
Pink	172	3.1	4.69	3.02	0.462	0.024	0.145
Subpop1	73	2.6	3.85	2.78	0.434	0.023	0.405
Subpop2	18	0.4	3.38	2.8	0.425	-0.004	0.918
Subpop3	16	5.3	3.46	2.93	0.461	-0.012	0.869
Subpop4	13	2.4	3.31	2.94	0.462	-0.005	0.972
Subpop5	62	3.7	3.92	2.94	0.462	-0.009	0.746
All	182	3	4.77	3.02	0.462	0.033	0.037

*n*, sample size; *NA*, number of alleles; *A*_R_(*k* = 14) allelic richness in sample of 14 gene copies (seven individuals); *H*_E_, gene diversity corrected for sample size); *F*_I_, individual inbreeding coefficient; *P*, probability that *F*_I_ is different from zero based on 10 000 permutations.

### Determinants of within-population genetic structure

Using MCMCglmm models with a single predictor matrix, we found that models with spatially defined matrices (GEO, ALT, or SUB) predicted inter-individual relatedness in the BDB population better, as shown by their lower DIC values than models including information on flower colour (COL) or vegetation at plant contact (VEG) (Table 4). This result suggests a stronger support for our Hypothesis 2 (spatial determinants of genetic structure) than for our Hypothesis 1 (flower colour affects genetic structure). Specifically, the GEO model had a lower DIC than the COL model, showing that spatial distance explained genetic relatedness better than corolla colour. Of all models compared, including those with multiple predictor matrices, the GEO_ALT model best predicted inter-individual relatedness (deltaDIC = 0). Predictive power remained similar when adding information on flower colour or substrate (GEO_ALT_COL or GEO_ALT_SUB).

**Table 4.** MCMCglmm modelling results for predicting inter-individual genetic relatedness.

Predictor variables	DIC	deltaDIC	DICweight
GEO_ALT	−25594.7	0	0.4044
GEO_ALT_SUB	−25594.6	0.11	0.3834
GEO_ALT_COL	−25593.5	1.29	0.2123
GEO	−25549.9	44.82	7.49E−11
GEO_COL	−25547.9	46.89	2.66E−11
ALT	−25423.1	171.66	2.14E−38
SUB	−25355.3	239.45	4.09E−53
NULL	−25344.3	250.48	1.65E−55
VEG	−25343.3	251.47	1E−55
COL	−25342.4	252.31	6.59E−56
COL_VEG	−25341.4	253.37	3.88E−56

Predictor variables listed include: NULL = null model, GEO = spatial distances, ALT = altitude differences, SUB = substrate differences (ravine vs. scree slopes/outcrops), COL = differences in corolla colour, VEG = differences with regard to vegetation at contact with the focal plant (with or without vegetation). For further details on model definitions, see Materials and Methods. The model with the lowest deltaDIC is the one that best explains genetic relatedness.

### Characterization of within-population genetic structure

**Figure 3.**
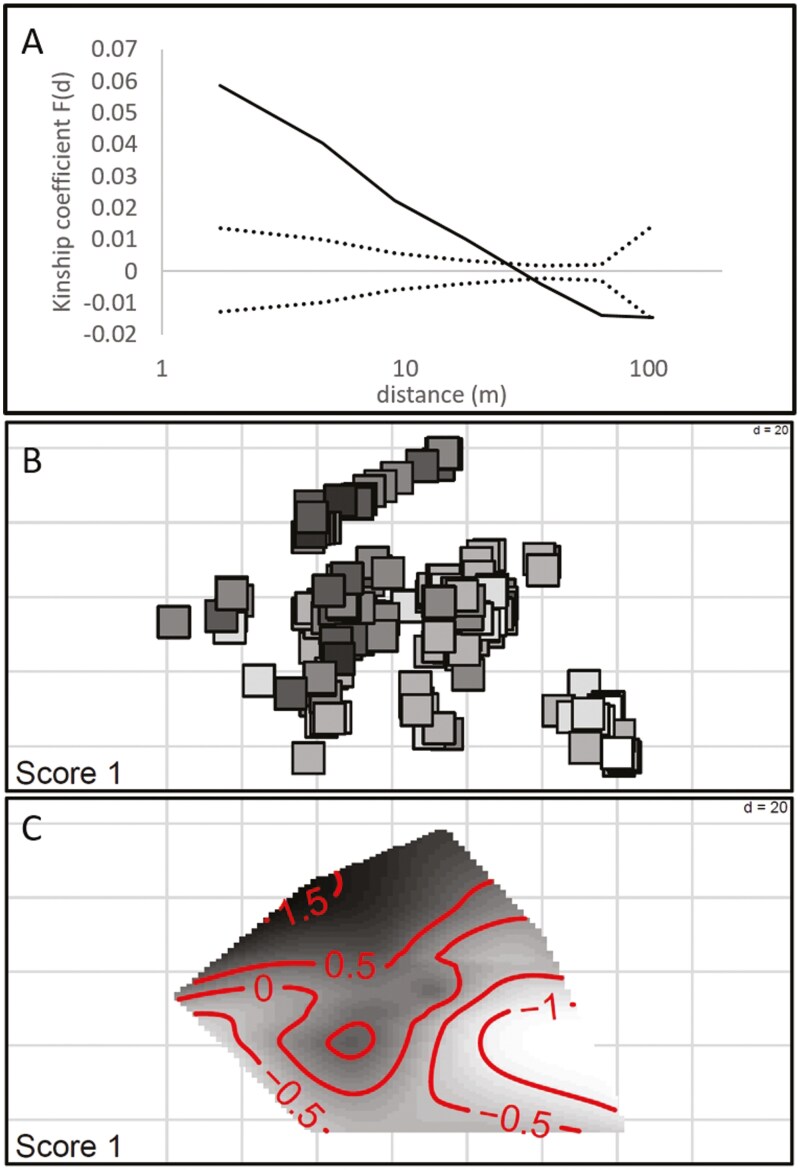
Isolation by distance pattern identified using nuclear SSRs. Kinship—distance regression; the 95% confidence interval for absence of isolation by distance is indicated with dotted lines (A). Individual scores on the first sPCA axis (B). Spatial interpolation of scores on the first sPCA axis (C).

Permutation tests revealed a significant decrease of inter-individual kinship as a function of the logarithm of distance for both pDNA and SSR data (*P* < 0.001 in both tests), with a markedly stronger spatial genetic structure at pDNA, *Sp*_p_ = 0.332 (see haplotype distribution in Fig. 2B), than at SSRs, *Sp*_n_ = 0.0198 ± 0.0001 SE (SSR kinship-distance plot in Fig. 3A). Spatial PCA confirmed the presence of a spatial genetic autocorrelation pattern through a significant *G*-test (*P* < 0.001), and revealed the absence of spatial genetic repulsion (non-significant *L*-test), with an eigenvalue of the first sPCA axis of eig.sPCA = 0.147. The geographic distribution of scores on the first sPCA axis is illustrated in Fig. 3B, where patches of similar shading indicate genetic similarity. Spatial interpolation of scores on the first sPCA axis is shown in Fig. 3C, revealing the spatial distribution and discontinuities in genetic similarity.

**Figure 4.**
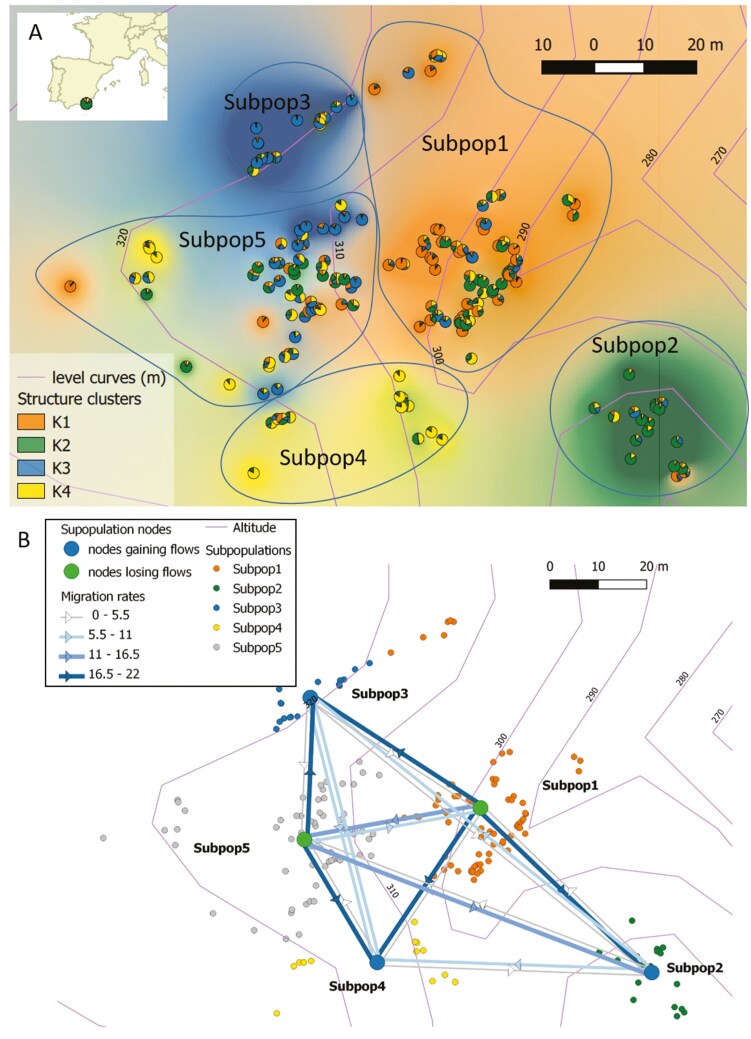
Genetic clustering based on STRUCTURE, for K = 4 clusters, and definition of subpopulations for Migrate analysis (outlined with continuous lines) (A). Graphic representation of migration rate estimates between subpopulations (MIGRATE program) using subpopulation definitions based on STRUCTURE analysis (B). Subpopulation nodes that are net sources of migrants are indicated in green; net sinks are indicated in blue (B).

Using the STRUCTURE software, *K* = 4 gene pools best explained the BDB data as this model had the highest lnP(P) and the highest deltaK [Supplementary Information—Fig. S4]. Spatial interpolation of cluster membership (Fig. 4) revealed the spatial clustering of related individuals, which led to the following definition of geo-genetic subpopulations (spatio-topographic groups of individuals, generally sharing genetic similarity): Subpop1, a large subpopulation dwelling in the narrow ravine gully in the eastern part of the population, where individuals were predominantly assigned to cluster K1 although other gene pools were also represented; Subpop2, a subpopulation on a rocky outcrop in the SE of the population with individuals mainly assigned to K2; Subpop3, a subpopulation on an ancient road cutting a scree slope in the north of the population, with individuals mainly assigned to K3; Subpop4, a subpopulation in the southern part of the population, representing mostly K4; and Subpop5, a large subpopulation corresponding to the uphill portion of the valley in the western part of the population, where all gene pools were represented (Figs. 2 and 4). GENELAND analyses [Supplementary Information—Fig. S5] were largely congruent with STRUCTURE analyses revealing a total of as much as *K* = 9 clusters, with larger patches of related individuals in the periphery of the population and a greater mixture in its centre, in the ravine.

The genetic differentiation between flower morphs was significantly different from zero with *F*_ST_ = 0.067. The white morph occurred in STRUCTURE gene pools 1, 2, and 4, and in subpopulations 1, 4, and 5, and the pink morph occurred in all gene pools and subpopulations. Differentiation among the four STRUCTURE gene pools was *F*_ST_ = 0.086, with pairwise *F*_ST_ ranging from 0.069 to 0.120 (Table 5). Differentiation among subpopulations was *F*_ST_ = 0.045; pairwise *F*_ST_ was the smallest, while significant, between the central Subpop1 and Subpop5, and the largest between peripheral Subpop2, Subpop3 and Subpop4 (maximum pairwise *F*_ST_ = 0.106, Table 5).

**Table 5.** Genetic differentiation (pairwise *F*_ST_) between colour morphs, between STRUCTURE gene pools, and between subpopulations defined based spatio-topographic position and STRUCTURE results. All values are significant with *P* < 0.0001.

Pink-white	0.068			
Gene pools	GP2	GP3	GP4	
GP1	0.069	0.079	0.101	
GP2		0.069	0.079	
GP3			0.120	
Subpopulations	Subpop2	Subpop3	Subpop4	Subpop5
Subpop1	0.045	0.079	0.065	0.020
Subpop2		0.106	0.071	0.063
Subpop3			0.095	0.047
Subpop4				0.052

### Inference of gene flow

In Migrate, the model with freely varying population parameters and migration rates was found to best explain the data, followed by the model with a single average migration rate and then the model with symmetric migration rates (Table 6). In the free model, theta estimates were similar for all subpopulations, with 1.45 ≤ Θ ≤ 1.79. The central subpopulations Subpop1 and Subpop5 were sources whereas the marginal subpopulations were mostly sinks for gene flow (Fig. 4B, Table 7). The number of migrants per generation received per subpopulation was always much larger than 1, a minimum rule-of-thumb threshold for conservation of genetic diversity (Mills and Allendorf 1996).

**Table 6.** Natural log marginal likelihoods (lmL) and natural log Bayes factors (LBF) for the tested migration modes.

Model	Thermodyn. lmL	Bezier lmL	LBF (Bezier)	Model prob.	Choice
free	−1160950.4	−187250.3	0.0	1	1 (best)
mean_m	−1312621.9	−211995.0	−49489.3	0	2
symmetric_m	−1811656.0	−291591.5	−208682.3	0	3
fixtheta_sym_m	−2878131.5	−461468.2	−548435.7	0	4
fixtheta_mean_m	−2886602.7	−462812.0	−551123.3	0	5

The thermodynamic and Bezier log marginal likelihoods are reported as given by the migrate software. For model definitions, see materials and methods.

**Table 7.** Migration rate estimates from Migrate software for a model with freely variable population parameters and migration rates.

	Receiving population					
Source population	Subpop1	Subpop2	Subpop3	Subpop4	Subpop5	Θ
Subpop1	0	21.6	19	19.3	14.6	1.45
Subpop2	1.6	0	9.6	9	2.2	1.68
Subpop3	1.6	4.1	0	8.2	2	1.54
Subpop4	1.6	6.9	5.5	0	2.1	1.79
Subpop5	6.4	15.8	19.7	15.9	0	1.52

Source populations for gene flow are represented on separate rows, receiving populations are represented in columns. The population parameter theta, Θ, is also represented.

For inference of gene dispersal distances from spatial genetic structure, we need to assume that the population is in drift-dispersal equilibrium. Despite evidence of population substructure and thus a likely violation of this assumption, inter-individual kinship decreased near-linearly with the logarithm of distance, and the iterative estimation of dispersal distance converged for both SSRs and cpDNA, resulting in an estimate of gene dispersal distance of *σ*_*g*_ = 21.707 meters and an estimate of seed dispersal distance of *σ*_*s*_ = 6.761 meters. The inferred ratio of seed-to-gene dispersal distance was *σ*_*s*_/*σ*_*g*_ = 0.311, inferred pollen dispersal distance was *σ*_*p*_ = 29.172 meters and the pollen-to-seed dispersal distance was *σ*_*p*_/*σ*_*s*_ = 4.315. These estimates should be interpreted with caution due to likely violation of assumptions, although the ratio estimates should be more robust.

## Discussion

In ecological and evolutionary contexts, the prevailing view is that pollinators have driven flower adaptations to novel pollinator niches since the origin of angiosperms (Rauscher 2008; Magallón and Vargas 2014; Peris and Condamine 2024). Within this framework, the *Antirrhinum majus*–*Bombus* system of flower–bee specialization has been proposed as an efficient driver of corolla evolution at the population level based on experimental (Dyer *et al.* 2007; Shang *et al.* 2011; Whitney *et al.* 2013) and field (Tastard *et al.* 2008) studies. However, over-generalizing the role of this pollination system in the evolution of any *Antirrhinum* species is problematic (see Romero *et al.* 2020). Indeed, most results focus primarily on the *Antirrhinum majus*-*Bombus terrestris* system, but they may not be representative of the interactions between other *Antirrhinum* species and their pollinators (Vargas *et al.* 2017). Accumulating evidence suggests that not only pollinator behaviour patterns but also spatial isolation have played crucial roles in the diversification of snapdragons (Vargas 2009; Otero *et al.* 2021; Durán-Castillo *et al.* 2022). Fine-scale analyses that concurrently examine both spatial and environmental factors are lacking in the field. Our results from *A. charidemi* contribute new insights into the relative contributions of these elements to the early stages of differentiation. We showed that in the topographically heterogeneous Barranco del Drangoncillo Blanco population, the genetic structure was best explained by a model that included topographic position only (latitude, longitude, and altitude). The strongest patterns of differentiation identified in the population corresponded to partially isolated peripheral groups of individuals. Insect visitors displayed a preference for pink over white flowers and flower morphs were significantly differentiated. However, flower morphs were not associated with inferred gene pools or any geographically defined subpopulations. Overall, our results suggested primarily contributions of both spatial and pollinator-mediated processes to genetic differentiation in this *A. charidemi* population, with a stronger role of the former.

### Bee diversity provides multiple pollination opportunities

Our characterization of flower visitors on *A. charidemi*, including ten bee and one beetle species, is in agreement with previous field results. (i) Bees are the most important insect guild for pollen and nectar collection in the genus *Antirrhinum* (Vargas *et al.* 2017), (ii) large bumblebees are not necessary to prise open the occluded corolla (but see Davies *et al.* 2006), and (iii) medium to large bees appear to be responsible for effective pollination (Vargas *et al.* 2010) (Table 1). In our study, we recorded ten bee species visiting *A. charidemi*, which represents one of the richest faunas of pollinators associated with *Antirrhinum* species (Vargas *et al.* 2017). *Antirrhinum* species are clustered into two pollinator niches with respect to the diversity and functions of their visiting pollinators. The first niche contains *Antirrhinum majus* and seven other *Antirrhinum* species mostly visited by large nomadic long-tongued bees (essentially of the genus *Bombus*). *Antirrhinum charidemi,* together with six more snapdragon species, forms part of the second niche, which exhibits a higher diversity of pollinator species including long-tongued nomadic, long-tongued territorial, and short-tongued bees (Vargas *et al.* 2017). A rich pollination fauna entails a high number of bee species from different taxonomic families, body sizes and shapes, and behaviour patterns (Vargas *et al.* 2017), which can bring about conflicting selection pressures in relation to pollen transfer (Lankinen and Larsson 2009).

The fauna of flower visitors in *A. charidemi* showed a significant preference for pink flowers over white flowers (Table 1). Only five of the ten bee species displayed a significant preference for a flower morph (three preferred pink, two preferred white), but these results at the species level hide strong differences in visitation rates by the different species. The two main pollinators, *Chalicodoma lefevrei* (441 visits, significant preference for pink) and *Rhodanthidium sticticum* (216 visits, non-significant test), represented 75% of flower visits observed (878 in total). Preference for pink flowers was also pronounced in two additional bee species with a robust sample size, *Anthophora dispar* (Apidae, large, nomadic) and *Ceratina cucurbitina* (Apidae, small, territorial), whereas significant preference for white colour relied on very small samples in two species, *Megachile deceptoria* and *Chalicodoma sicula* (Table 1). To assess whether such flower colour preferences contribute to genetic differentiation, it remains to be tested whether flower visits by these species lead to effective pollen deposition and seed set in the visited flowers. Further analyses at the species level, coupled with that at the individual level, over time might reveal even greater complexity in flower visitor behaviour between the two colour morphs.

The insect guild visiting *A. charidemi* flowers illustrates that bee movements could be significantly different among species and individuals. The two main flower visitors are Megachilidae species (*Rhodanthidium sticticum* and *Chalicodoma lefevrei*) which display foraging behaviours and pollination systems considerably different from those of *Bombus* species (typical of the *A. majus* pollinator niche). *Bombus* individuals deposit pollen by brooming into a specialised leg structure (corbicula); instead, Megachilid bees broom and deposit pollen onto the ventral surface of the abdomen (scopa) (Michener 1999). Pollination can also be different within the same bee species. For example, the territorial behaviour pattern observed for males of *Rhodanthidium sticticum* potentially results in more spatially restricted pollen flow than in drones or queens of nomadic *Bombus* species (see Fragoso and Brunet 2023). Conversely, females of *R. sticticum* showed nomadic movements among plants, which prevented from considering a single behaviour pattern for each bee species (Romero *et al.* 2020). Interestingly, short-distance gene flow by pollen mediated by *R. sticticum* males may be reinforced by the observed territorial, small bees (*Ceratina cucurbitina*) and the social honeybees (*Apis mellifera*), which have preference for floral constancy and pollination of nearby plants (Vargas *et al.* 2010; P. Vargas personal observation)

### Fine-scale spatial isolation by topography

We observed a strong effect of topography on genetic structure in the Barranco del Dragoncillo Blanco population, where the combination of spatial distance and terrain differences (altitude) were the strongest predictors of genetic structure (Table 4). This strong isolation signal is remarkable given the small population size (ca. 120 × 80m, e.g. about 1 ha), which is significantly divided into four-five distinct subpopulations (Fig. 4). Topography, particularly substrate type (classified as rock, wall, or soil), also played a crucial role in the differentiation of two additional *Antirrhinum* species endemic to south-eastern Spain (Barnbrook *et al.* 2023). In a population of the snapdragon *A. microphyllum* inhabiting rugged terrain in central Spain, Torres *et al.* (2003) also identified clusters of genetically similar individuals within small patches. To assess whether such differentiated subpopulations may represent early stages potentially leading to speciation, it would be interesting to examine their genomic patterns of divergence to search for any signatures of adaptive divergence.

The fine-scale spatial genetic structure observed in our population *Sp*_*n*_ = 0.020 ranks in the upper 30% of species with the strongest spatial structure and was typical for plants with gravity-dispersed seeds (mean *Sp* = 0.028) and animal-dispersed pollen (mean *Sp* = 0.017, Vekemans and Hardy 2004). Our estimate of the pollen-to-seed dispersal distance ratio of 4.31 m suggests a strong effect of gravity-mediated seed dispersal which contributed to isolating groups of tens of individuals on a microspatial scale (1 ha). Our estimated gene dispersal distance was *σ*_*g*_ = 21.7 meters, pollen dispersal distance *σ*_*p*_ = 29.2 meters, and gene flow via seed dispersal distance *σ*_*s*_ = 6.8 meters. These values should be interpreted with caution due to likely model violations. However, they appear further-ranging than estimates in other plants with similar or stronger *Sp*, e.g. *Silene acaulis*, *Medicago trunctula,* or *Hibiscus moscheutos*, which have gene dispersal estimates, σ_g_, that do not exceed 5 metres. The lower effective density of *A. charidemi* compared to those species can at least partially explain this difference (see Vekemans and Hardy 2004). In summary, topography appears to be key for microevolutionary patterns of differentiation in *Antirrhinum charidemi*. This result is congruent with that of Marrot and collaborators (2022), who found that natural selection acting on several vegetative traits varied both in magnitude and in direction in response to environmental variables at the fine spatial scale of even a meter.

Isolation by distance has played a main role in differentiation also at larger scales. Phylogeography and genetic diversity of *A. charidemi* across the narrow volcanic mountains of Almería revealed a strong effect of geographic isolation on the population genetic structure (Forrest *et al.* 2017). At a biogeographic scale, spatial isolation leading to speciation is also interpreted because *A. charidemi* exhibits a well-supported phylogenetic association with two species (*A. mollissimum*, *A. rupestre*) also endemic to south-eastern Spain (Otero *et al.* 2021; Durán-Castillo *et al.* 2022).

## Conclusions

Our population-scale study in the narrow endemic *Antirrhinum charidemi* identified a preference of most flower visitors for the pink flower morph, along with a significant genetic differentiation between the pink and white flower morphs. However, fine-scale spatial isolation in a topographically heterogeneous environment was the strongest driver of genetic differentiation in our study. These results on the very local scale support the hypothesis of spatial isolation as a prime force for evolutionary divergence in *Antirrhinum* previously verified on the biogeographic (macroevolutionary) scale and on the intra-specific (microevolutionary) scale in some species. We consider that spatial isolation followed by particular reproductive systems (self-incompatibility, exclusive reliance on bee pollination, pollen gene flow dependent on bee behaviour patterns, unassisted seed dispersal) and additional ecological conditions (topography, climate, substrate types, and altitude) have been particularly determinant for the microevolutionary patterns found in *A. charidemi*. Further research quantifying how spatial and ecological factors interact to drive differentiation of each *Antirrhinum* species is needed to determine their relative contributions in the early stages of differentiation.

## Supplementary Material

plaf017_suppl_Supplementary_Materials

## Data Availability

The data matrix with microsatellite and plastid DNA genotypes as well as metadata is available on Zenodo (https://doi.org/10.5281/zenodo.14198421). Flower visitor data is found in Table 1.
