# Supplementary material for: Fine-scale genetic differentiation in the bee-specialized Antirrhinum charidemi covaries more strongly with spatial isolation than with corolla colour
Source: AoB Plants. 2025 Mar 24;17(4):plaf017. doi: 10.1093/aobpla/plaf017 (PMC12285732; doi:10.1093/aobpla/plaf017)

Supporting information to

Fine-scale genetic differentiation in the bee-specialized Antirrhinum charidemi covaries more strongly with spatial isolation than with corolla colour

Myriam Heuertz, Marcial Escudero, José María Gomez & Pablo Vargas

Table S1. Results of the permutation-based multivariate analysis of variance (PERMANOVA) for the two corolla morphs in *Antirrhinum charidemi* (pink and white) indicating that they are visited by bee species in similar relative frequencies.

	Df	Sum of Squares	R_2	*F*	*P* value
Floral Morph	1	0.244	0.023	0.463	0.976
Residual	20	10.549	0.977		
Total	21	10.794	1.000		

Table S2. Comparison of estimated null allele frequencies using four algorithms in *Antirrhinum charidemi*, based on Micro-Checker version 2.2.3.

Locus	Repeat type	Null Present	Oosterhout	Chakraborty	Brookfield 1	Brookfield 2
Ant11AM2	Di	no	0	0	0	0.2236
MSAT13	Di	no	0	0	0	0.322
MSAT77	Di-tetra	no	0.0083	0.0086	0.0026	0.0655
MSAT71	Di	no	0	0	0	0.6124
MSAT26	Tri	no	-0.0307	-0.0272	-0.0104	0
MAT4	Tetra	no	0	0	0	0.9739
AN1	Di	yes	0.0614	0.0562	0.0391	0.1025
MSAT19	Di	no	0.0765	0.2394	0.0162	0.2872
MSAT55	Tri	no	-0.012	-0.0124	-0.0103	0
MAAC4	Di	no	0	0	0	0.2661
MSAT63B	Tri	yes	0.0644	0.0676	0.0486	0.0486
MSAT44	Di	yes	0.1369	0.2075	0.0696	0.2887
MSAT69	Di	no	-0.018	-0.0155	-0.0139	0.0231

Table S3. Genetic diversity statistics of 9 SSR markers (excluding AN1, MSAT63B and MSAT44 with low-frequency null alleles, and MAAC4 to correct for LD) for flower-colour based categories, and topographic-genetic groups based on the STRUCTURE analysis in *Antirrhinum charidemi*. n, sample size; NA, number of alleles; AR (k=14) allelic richness in sample of 14 gene copies (seven individuals); HE, gene diversity corrected for sample size); Fi, individual inbreeding coefficient; P, probability that FI is different from zero based on 10,000 permutations.

Category	n	missing %	NA	A_R(k=14)	H_E	F_I	P
white	10	1.10%	2.56	2.4	0.345	-0.022	0.780
pink	172	3.60%	4.22	2.88	0.450	0	0.900
Subpop1	73	3.00%	3.44	2.69	0.415	-0.029	0.367
Subpop2	18	0.60%	3	2.58	0.392	-0.038	0.562
Subpop3	13	2.60%	3	2.74	0.446	-0.04	0.582
Subpop4	16	7.60%	3.22	2.9	0.457	0.043	0.523
Subpop5	62	3.90%	3.67	2.83	0.460	-0.014	0.728
All	182	3.50%	4.33	2.87	0.448	0.008	0.740

Table S4. Genetic differentiation (pairwise F_{ST}) between subpopulations defined based on STRUCTURE results, using 9 SSR loci (excluding AN1, MSAT63B and MSAT44 with low-frequency null alleles, and MAAC4 to correct for LD) in *Antirrhinum charidemi*. All values are significant with P<0.0001.

	Subpop2	Subpop3	Subpop4	Subpop5
Subpop1	0.040	0.067	0.061	0.017
Subpop2		0.088	0.116	0.068
Subpop3			0.102	0.060
Subpop4				0.040

Figure S1. Results of a spectrophotometric analysis of flower colouration in *Antirrhinum charidemi* represented using a colour space model. Four hexagons are used for four individuals modelling bee vision: two with only pink and two with only white corollas. For each plant individual 10 flowers where measured, including four measurements per flower.

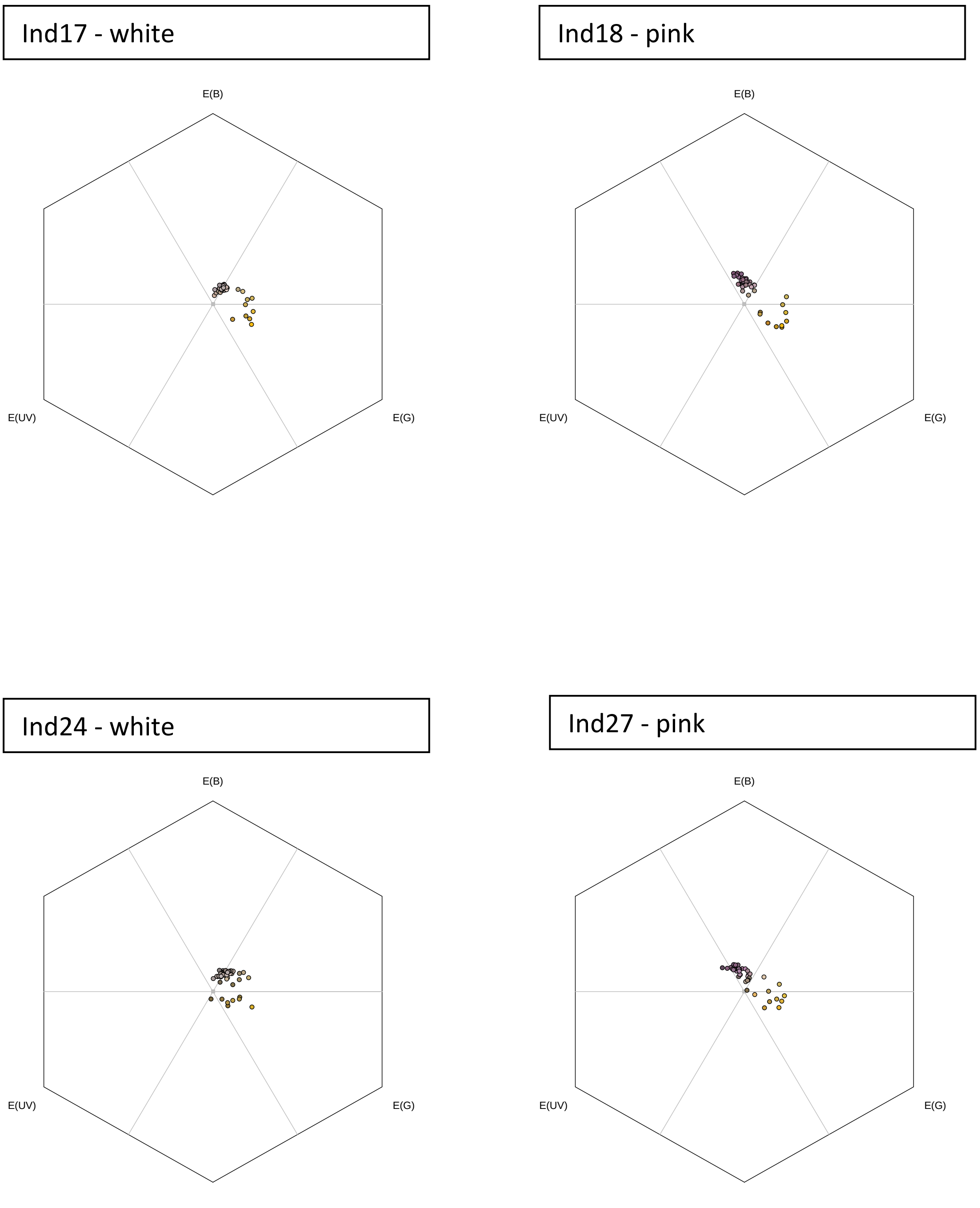

Figure S2. Results of the Jacob´s D index of preference for every potential pollinator visiting *Antirrhinum charidemi*: nine bees and one beetle (*Oxythyrea funesta*). Dots in red indicate significant values; dots in grey non-significant values.

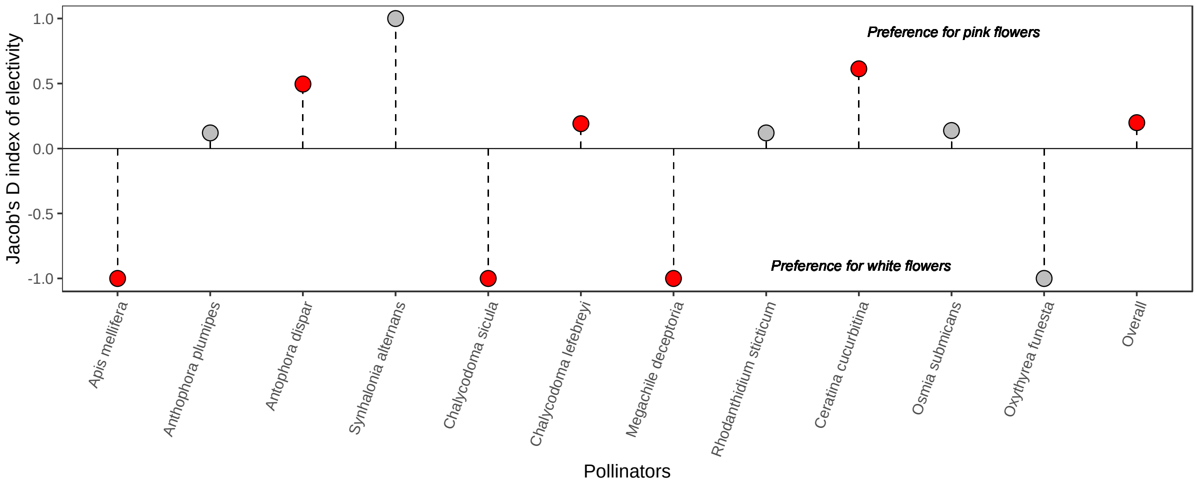

Figure S3. Proportion of corolla colour (pink or white) preference by the five main bee potential pollinators of *Antirrhinum charidemi*. Rsti, *Rhodanthidium sticticum*; Osub, *Osmia submicans*; *Calycodoma lefebreyi*; Ccuc, *Ceratina cucurbitina*; Adis, *Anthophora dispar*.

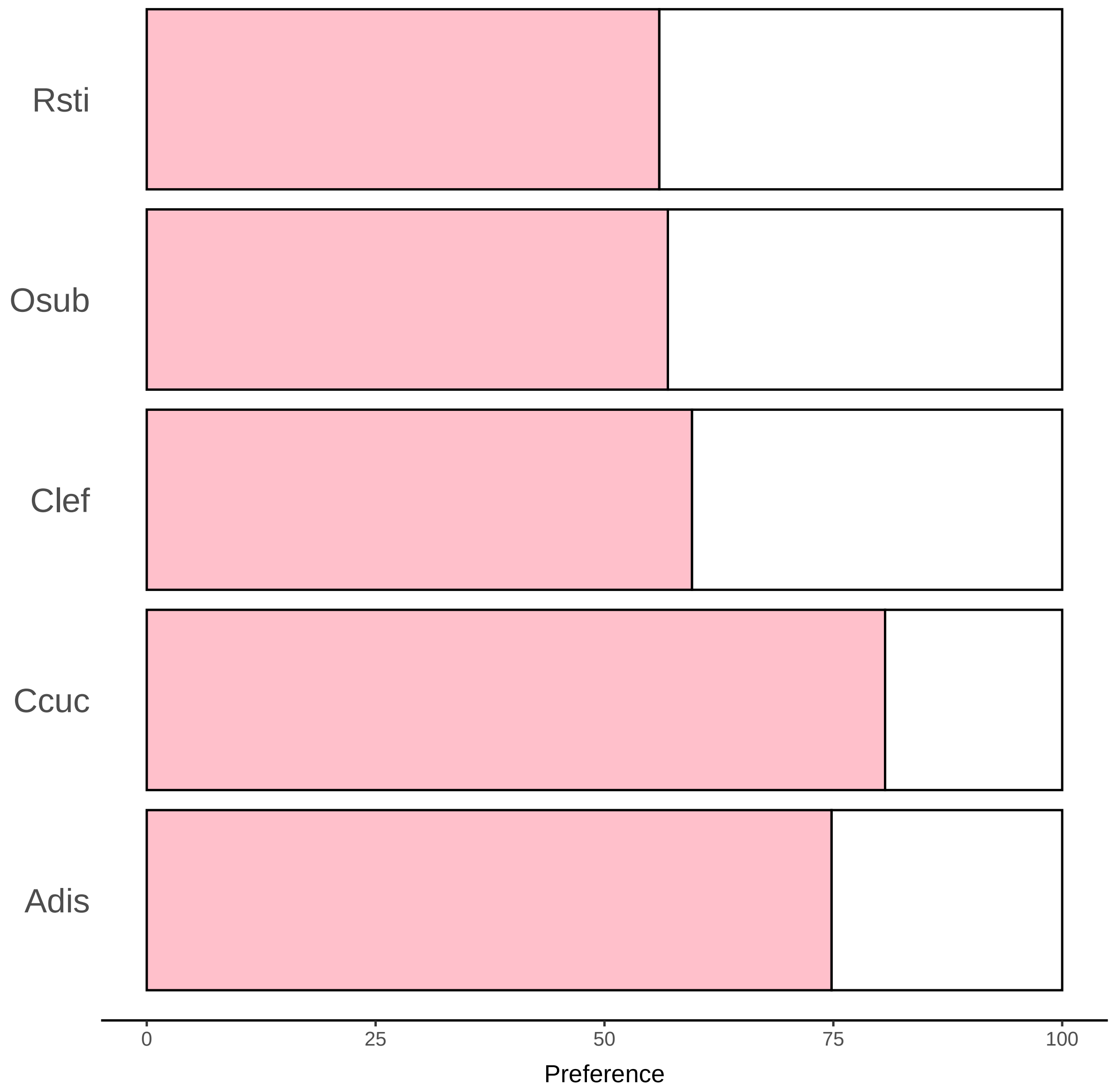

Figure S4. Choice of the best number of clusters in a STRUCTURE analysis of SSR data in *Antirrhinum charidemi.* A. Mean logarithm of the probability of data given K clusters in a STRUCTURE analysis. B. Delta K method for Evanno and colleagues, 2005. K=4 is the most likely number of clusters, results shown in the paper.

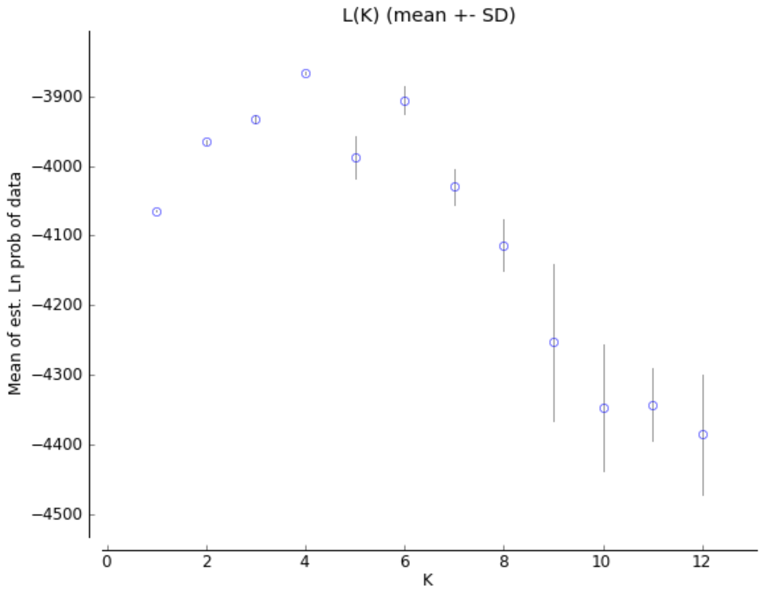

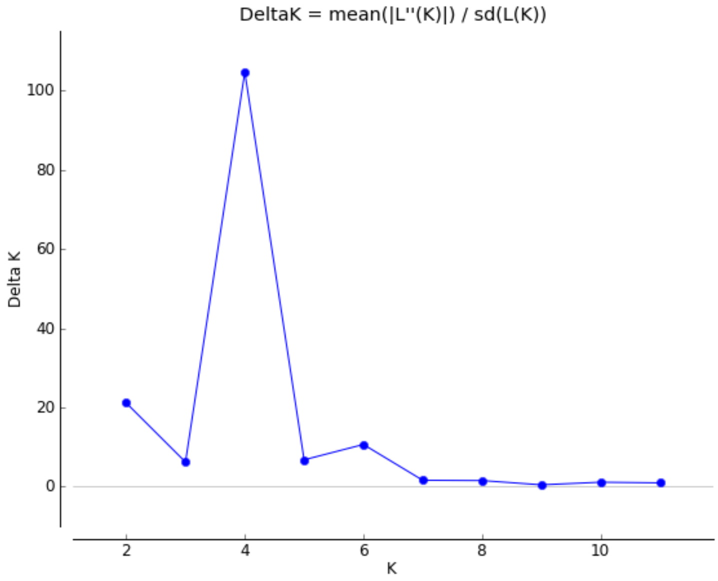

Figure S5. Genetic clustering obtained in a GENELAND analysis of SSR data in *Antirrhinum charidemi*, for K=9 clusters.

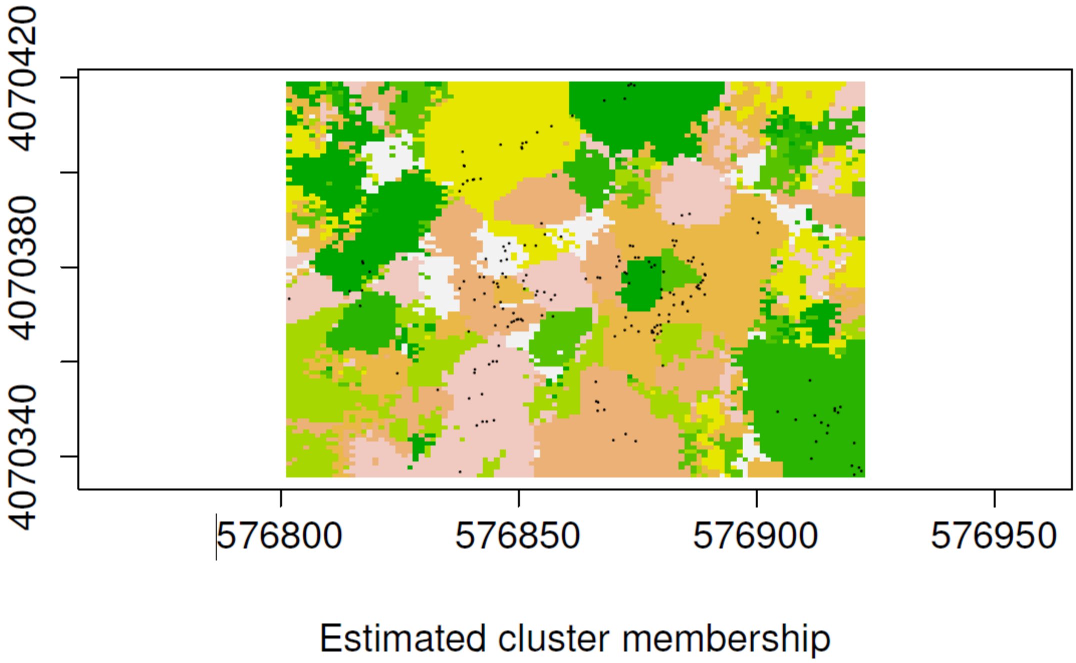

Supplement: plaf017_suppl_Supplementary_Materials [file plaf017_suppl_supplementary_materials.pdf]
